# Vision Intervention for Seeing Impaired Babies: Learning through Enrichment (VISIBLE) – protocol of a feasibility pilot randomised controlled trial

**DOI:** 10.1136/bmjopen-2025-114567

**Published:** 2026-04-20

**Authors:** Andrea Guzzetta, Ada Bancale, Anna Bedoshvili, Margot Bosanquet, Olena Chorna, Giulia Corsi, Sabrina Del Secco, Catherine Elliott, Simona Fiori, Jurgen Fripp, Glen A Gole, Anya Gordon, Karen Harpster, Rod W Hunt, Shaneen Leishman, Rosalie Mori, Catherine Morgan, Iona Novak, Alex Michael Pagnozzi, Kerstin Pannek, Swetha Sara Philip, Melissa Rice, Alison Salt, Bernadette Shannon, Terry Schwartz, Nana Nino Tatishvili, Sofia Tatishvili, Roslyn N Boyd

**Affiliations:** 1 Department of Clinical and Experimental Medicine, University of Pisa, Pisa, Italy; 2 Department of Developmental Neuroscience, IRCCS Stella Maris, Pisa, Italy; 3 David Tvildiani Medical University, Tbilisi, Georgia; 4 Bokeria Neurodevelopmental Center, Tbilisi, Georgia; 5 Department of Health and Wellbeing, Townsville University Hospital, Douglas, Queensland, Australia; 6 School of Medicine, University of Queensland, Herston, Queensland, Australia; 7 School of Medicine and Dentistry, James Cook University, Douglas, Queensland, Australia; 8 NEUROFARBA, University of Florence, Florence, Italy; 9 School of Allied Health, Curtin University, Perth, Western Australia, Australia; 10 Telethon Kids Institute, Nedlands, Western Australia, Australia; 11 Neuroscience and Medical Genetics Department, Meyer Children’s Hospital IRCCS, Florence, Italy; 12 The Australian E-Health Research Centre, CSIRO, Brisbane, Queensland, Australia; 13 CHQ Clinical Unit -UQ, Queensland Children's Hospital, Brisbane, Queensland, Australia; 14 Queensland Cerebral Palsy and Rehabilitation Research Centre, The University of Queensland, Brisbane, Queensland, Australia; 15 Occupational Therapy and Physical Therapy, Cincinnati Children’s Hospital Medical Center, Cincinnati, Ohio, USA; 16 Pediatrics, University of Cincinnati College of Medicine, Cincinnati, Ohio, USA; 17 Paediatrics, Monash University, Clayton, Victoria, Australia; 18 Physiotherapy, King Edward Memorial Hospital for Women Perth, Subiaco, Western Australia, Australia; 19 Cerebral Palsy Alliance Research institute, Specialty of Child and Adolescent Health, Faculty of Medicine and Health, The University of Sydney, Sydney, New South Wales, Australia; 20 The University of Sydney Faculty of Medicine and Health, Sydney, New South Wales, Australia; 21 The University of Queensland, Brisbane, Queensland, Australia; 22 Division of Ophthalmology, Cincinnati Children’s Hospital Medical Center, Cincinnati, Ohio, USA; 23 Department of Ophthalmology, University of Cincinnati College of Medicine, Cincinnati, Ohio, USA; 24 Paediatric Rehabilitation, Perth Children’s Hospital, Nedlands, Western Australia, Australia; 25 University of Western Australia, Perth, Western Australia, Australia; 26 Neuroscience Department, M.Iashvili Central Children’s Hospital, Tbilisi, Georgia

**Keywords:** Brain, Randomized Controlled Trial, Developmental neurology & neurodisability, Neurological injury, NEONATOLOGY

## Abstract

**Introduction:**

Visual impairment is reported to affect 40%–50% of children with cerebral palsy (CP). Vision difficulties in the context of rehabilitation are often under-recognised, under-treated and therefore under-studied, pointing to an urgent need for the development of evidence-based vision interventions for infants and toddlers with cerebral vision impairment (CVI). We present the protocol of a multisite pragmatic pilot randomised controlled trial (RCT) of feasibility, acceptability and preliminary efficacy of an early vision-awareness and parent-directed environmental enrichment programme for infants with or at risk of CP under 7 months corrected age (CA) with vision impairment.

The main objective is to determine the feasibility and acceptability of the Vision Intervention for Seeing Impaired Babies: Learning through Enrichment (VISIBLE) intervention. We will estimate the preliminary effects of the programme on infants’ visual functions and early development, as compared with standard community-based care (SCC).

**Methods and analysis:**

A two-group RCT will be conducted. Infants at 3–6 months at entry, with severe visual impairment and at high risk of CP, will be enrolled and randomised (n=16 per group) to receive the VISIBLE intervention compared to SCC. Randomisation will be completed through an independent automated process (Research Electronic Data Capture). VISIBLE intervention will be delivered by a therapist through home visits (90–120 min) once every 2 weeks. Completion of 10 visits (80% of the intervention target dose) within 6 months is required for adherence to the VISIBLE trial. Outcome will be assessed at 12 months CA. Visual function will be evaluated with the Infant Battery for Vision, motor outcomes with the Peabody Developmental Motor Scales, Second Edition. Developmental quotients, infant quality of life, parent well-being and parent-infant relationship will be also monitored through standardised tools.

**Ethics and dissemination:**

The enrolling sites have historically demonstrated rapid and effective translation of successful evidence-based interventions into routine clinical practice, as well as the dissemination of the findings through local, national and international scientific meetings.

**Trial registration number:**

ACTRN12618000932268.

**STRENGTHS AND LIMITATIONS OF THIS STUDY**

- This pilot feasibility randomised controlled trial addresses a gap in rehabilitation interventions for infants with cerebral palsy (CP)/high risk of CP and severe visual impairment.
- The home-based programme of vision-awareness and parent-directed environmental enrichment programme will be supported by a multidisciplinary team through parent training and fortnightly home visits.
- The daily goal-oriented activities provided by the parents focus on vision-aware developmental goals and environmental enrichment.
- The recruiting sites (Pisa, Italy; three sites in Queensland, Australia; Western Australia; Tbilisi, Georgia; Cincinnati, Ohio, USA) use existing early detection and intervention networks to identify and recruit families.

## Introduction

### Background

 Visual impairment in children with cerebral palsy (CP) is very common. While about 10% of the children with CP present with a severe form of visual impairment (inability to fix and follow a target),^1^ milder levels of visual perceptual impairments have been reported in an additional 40% of them.^2^ Visual difficulties in children with CP can be related to disorders of the anterior visual pathways or ocular impairment, such as retinopathy of prematurity or severe refractive error, but most often they are due to damage to the post-chiasmatic visual pathways or the visual cortex, leading to cerebral visual impairment (CVI).^3 4^

Visual impairment can have a huge impact on development.^5^ Neurodevelopmental outcomes are highly dependent on the infant’s experience and interaction with the environment, especially their parents or caregivers.^6^ The ability to visualise the social and physical environment, including caregivers, is essential to infant development, as it provides input and feedback for the integration of all sensory channels so that the infant can make sense of their world.^7^ Early visual impairment profoundly affects multiple aspects of development. In motor development, vision plays a crucial role in guiding movement, spatial awareness and coordination, so its absence can lead to delays in reaching, grasping, crawling and walking.^8^ Cognitive development is also impacted, as vision is essential for learning, memory formation and problem-solving.^9^ Furthermore, social interactions and emotional regulation can be impacted, as visual cues such as facial expressions, gestures and eye contact are vital for communication and emotional bonding.^10^ The effect of visual impairment on development is further amplified in infants with brain damage, as they may show dysfunctions in other integrated sensory and motor functions.

In spite of the high prevalence of CVI in children with CP and a consensus on the need for early detection of visual function impairment,^11 12^ the role of vision in the context of rehabilitation is under-recognised, under-treated and under-studied. In infants with severe vision impairment, early intervention services need to be used more effectively to include child’s vision abilities within the design of individualised intervention strategies.^13^

While guidelines for the diagnosis and referral of CVI have been developed,^14^ providing an evidence-based pathway to guide clinicians through the diagnostic process, no such guidelines exist for intervention in infants with CVI. A recent systematic review of interventions for children with CVI found insufficient evidence to support evidence-based clinical practice, primarily due to the low level of evidence and small sample sizes in the analysed studies.^15^ Nevertheless, encouraging findings have emerged. Fazzi *et al*^16^emphasized the importance of early visual training and environmental adaptations in infants aged 4–11 months, demonstrating their potential to enhance functional vision and neurodevelopmental outcomes, particularly in those with cerebral palsy. Similarly, Cemali *et al*^17^ showed that an 8-week sensory integration intervention, conducted twice weekly, significantly improved motor and sensory functions in infants with CVI and cerebral palsy at 12–18 months. While emerging studies highlight promising results, limited evidence exists in the first year of life as no randomised controlled trial (RCT) addressed specific interventions in infants with CVI in this early stage of development.

In infants with severe vision impairment, early intervention should include child’s vision abilities within the design of individualised intervention strategies.^13^ We have defined this as a vision-aware therapeutic approach, where the definition of awareness is twofold. Full awareness of child’s functional vision is required from the healthcare professionals as indispensable knowledge for optimal designing of the individualised treatment plan. At the same time, the visual adaptations provided as part of the intervention are necessary to increase child awareness of their own visual capabilities, further fostering functional use of vision in everyday experience. Consistent with these premises and with recently published international guidelines on early intervention in infants with brain damage,^12^ we developed an early vision-aware and parent-directed environmental enrichment programme called VISIBLE (Vision Intervention for Seeing Impaired Babies: Learning through Enrichment).^12 18^ Through a multisite pragmatic pilot RCT study of the VISIBLE programme, we will determine its feasibility and acceptability. Should the study show initial evidence of efficacy, the data will be used to design a new and adequately powered RCT.

### Aims

1. To determine the feasibility and acceptability of the VISIBLE intervention programme for infants with severe CVI and high risk of cerebral palsy compared with standard community-based care (SCC) in a randomised controlled trial.
2. To evaluate preliminary efficacy of the VISIBLE programme to lead to greater improvements of (i) visual function, (ii) developmental outcomes (developmental, gross motor, fine motor) and (iii) parent-infant emotional and relational development, as compared with standard of care (SoC).

### Major hypothesis

Our main hypothesis (H1) is that infants with severe CVI and high risk of cerebral palsy who receive the VISIBLE programme will have superior visual behaviour at 12 months CA compared with those who receive SCC.^19^

### Secondary hypotheses

H2 Infants with severe CVI and high risk of CP who receive the VISIBLE programme will have superior motor performance at 12 months CA and superior cognitive scores on standardised assessments at 12 months CA, compared with those who receive SCC.^9 20^

H3 Parents will find the VISIBLE programme intervention feasible, measured via recruitment rates and intervention adherence, acceptability measured by rates and reasons for withdrawal and high engagement scores and satisfaction.

## Methods and analysis

### Trial design

This is a multisite pragmatic RCT pilot study of early vision-aware and parent-directed environmental enrichment programme (VISIBLE).

### Participants

Infants will be identified from neonatal follow-up programmes and early detection networks in Pisa, Italy (Stella Maris Research Institute and University of Pisa), Queensland, Australia (Queensland Cerebral Palsy and Rehabilitation Research Centre at the University of Queensland), Western Australia, Australia (Perth Children’s Hospital), Tbilisi, Georgia (David Tvildiani Medical University) and Cincinnati, USA (Cincinnati Children’s Hospital Medical Center). Children meeting inclusion criteria for VISIBLE will be invited to participate.

We will accept referrals from families located close to or remotely from the recruiting centres if the family agrees to attend at least 50% of the assessment visits at the medical centre. If an enrolled family lives >1 hour travel distance or home visits are otherwise difficult, teleconferences will be organised to perform up to 50% of the intervention sessions. The study started in July 2018 and is anticipated to continue until December 2027.

Acquisition of informed consent: A member of the research team explains the project to parents and provides written information about the study so that it can be read and understood in their entirety. An example of the consent form is included as an online supplemental file 1. Parents have time to review the information, and the opportunity to ask any questions about the study. Parents are told that they can have more time to think about their willingness to participate in the study. In these cases, there is a subsequent conversation in which the parents are able to ask clarifying questions and definitively confirm their consent to the study.

**Figure 1:**
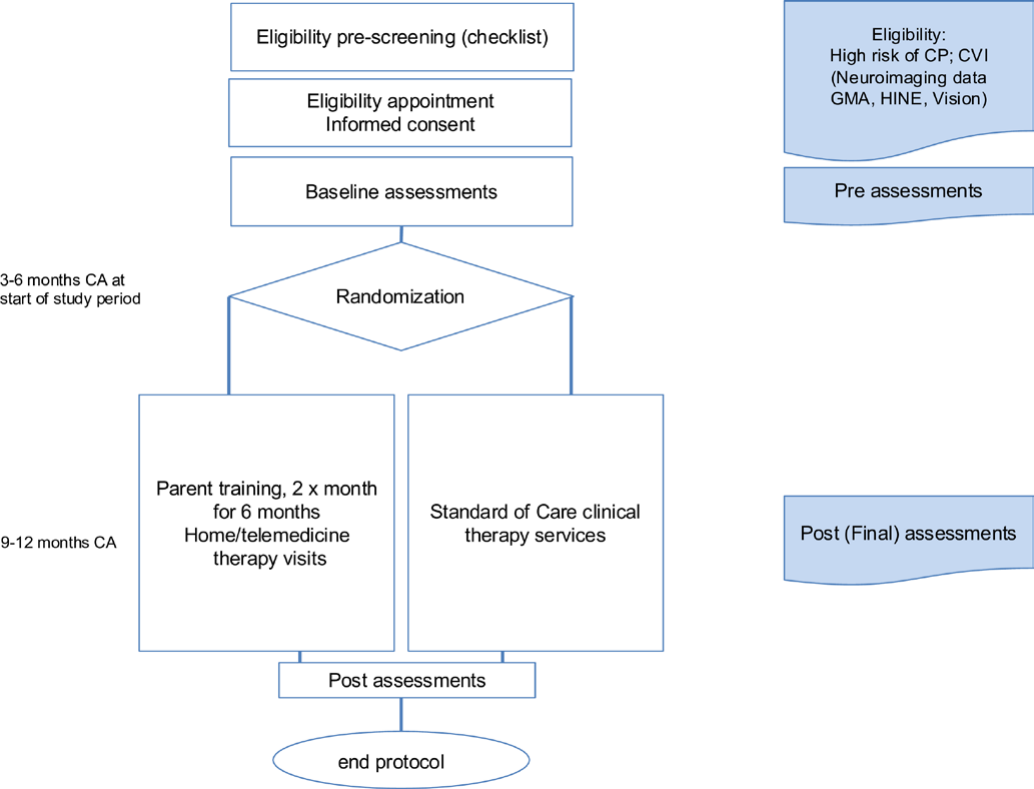
Study process flow. CA, corrected age; CP, cerebral palsy; CVI, cerebral vision impairment; GMA, General Movements Assessment; HINE, Hammersmith Infant Neurological Examination.

Individual written informed consent is obtained from parents of all participants in the study. Interpreters are sought for families who speak languages other than the locally spoken language/s. Figure 1 outlines the process flow of the protocol.

#### Inclusion criteria

1. Visual impairment (visual acuity values below 2 SD for their corrected age), based on visual acuity assessment using Teller Acuity Cards half set, consisting of 8 cards. ^21^They represent modulated gratings (between 0.23 and 26 cycles/cm) to measure maximum acuity. Distance of 38 cm is used for infants 0–6 months to allow for testing of spatial frequencies. This wide range is suitable for acuity measurement in infants with low vision;
2. A diagnosis of CP or of ‘high risk of CP’ per the International Clinical Guideline for Accurate Early Detection criteria^12 22^;
3. An age at recruitment under 7 months post-term (corrected age, CA).

#### Exclusion criteria

1. Infants with a medical fragility preventing the ability to participate in the activities;
2. Anatomical malformations preventing any residual vision (eg, bilateral anophthalmia or microphthalmia);
3. Drug-resistant epilepsy per the International League Against Epilepsy criteria.^23^

### Interventions

VISIBLE is a novel early intervention programme based on the core principles of optimising infant’s visual experience during the first phases of development.^13 16^ The general principles are activity-dependent learning and environmental enrichment, with parents and caregivers as the most essential element of environmental enrichment for infant learning success.^18^ They provide the environmental cues for both social and physical infant exposure. Using this principle, parents will provide vision-aware environmental enrichment—adapting the social and physical environment to allow the infant the opportunities to learn by successfully experiencing their enhanced environment. Within the context of parent-child daily goal-oriented interactions, environmental enrichment, as it relates to vision-aware modifications, will include light, spatial distances, salience, environmental consistency and multimodality of sensory experience.

Additional specific aspects of the VISIBLE programme for infants in the experimental group will include strategies of optimising infant’s visual experience and integration of vision with the other sensory channels. Parents will be coached on goal-directed vision-aware strategies to promote learning, movement and social interaction. Parent-infant interactions will be adaptive, supportive and consistent. Training through developmentally appropriate play will be conducted at the limit of the infant’s performance ability with variation and increasing complexity built into the play activities. The sessions will target infant’s specific developmental goals (including motor, communication, social-emotional and sensory) with an addition of the vision-aware strategies and will actively engage parents in providing daily functional activities.

The home-based programme of goal-directed vision-aware early intervention will be supported by a multidisciplinary team through parent training and fortnightly home visits (Direct or via Telehealth). The daily intervention activities will be primarily delivered by the parents, but supported by the VISIBLE therapist, and will focus on environmental enrichment and targeted vision-aware developmental goals. Study-specific materials have been developed to assist the parents in providing the intervention activities to their children.

#### VISIBLE

Within the first year of life, which is the target age for VISIBLE training in this pilot RCT, two main phases are identified that represent two sequential stages of development. The first phase is centred on the establishment of the parent-infant relationship and starts from birth. In this phase, it is essential for the child to have periods of quiet wakefulness to foster the development of the attachment relationship with the caregivers and to fully use their visual potential. The overarching goal of the intervention in this phase is to increase and improve the opportunities of positive dyadic interaction between the baby and the caregiver. The second phase is centred on the development of intentional actions and exploration. It layers on the first phase and typically begins at an age between 4 and 8 months CA, depending on the severity of brain damage and CVI. In this stage, the child has started to internalise the reference figures (parents, caregivers) and begins to show an increased interest in the surrounding environment, making more consistent attempts to organise goal-directed movements. The overarching goal of the intervention in this phase is to guide the infant in environmental exploration, through shared and infant-led activities.

##### Standard community-based care (SCC)

The SCC sessions are heterogeneous and are typically administered in clinics or rehabilitation settings. Some clinicians may focus on vision screening and visual strategies, whereas others will not. The therapy approaches and frequencies are variable. We expect that SCC intervention will vary significantly between participants in the study, both based on the family location and infant needs. We will ask for the parents in both groups to fill out a study-specific questionnaire to record the SCC their child is receiving, including the type of therapy, frequency and duration of sessions.

### Programme implementation

The project will be led and supported by multidisciplinary teams at each institution, including paediatric neurologists/rehabilitation specialists, paediatric ophthalmologists/optometrists, paediatric developmental therapists, paediatric physiotherapists and occupational therapists.

The initial eligibility confirmation and baseline assessments will take place at the respective recruiting centre. Medical needs and treatable eye problems (refractive correction, other preventative measures) will be addressed prior to the onset of the study period by a Paediatric Ophthalmologist/Optometrist for infants in both groups. On confirmation of eligibility and completion of baseline assessments, the family will be randomly allocated to SCC or VISIBLE.

For the intervention group, appointments every 2 weeks will be provided by VISIBLE-trained developmental therapists to contextualise the programme to the infant’s natural environment and support parents with implementation. The first home visit for the VISIBLE group families will include parent training and information about and demonstration of activities appropriate for their infant. The following visits (90–120 min duration) will include direct activities with the parents and infant, coaching the advancement of activities to accommodate the infant’s emerging skills and parent treatment fidelity checks. The information discussed during the visits will be also available to the parents in the form of a study-specific parent booklet. It consists of modules that will be provided to the parent based on the needs of the infant and the family.

#### Fidelity

We will use the model of fidelity recommended by the US National Institute of Health Behaviour Change Consortium, which^1^ comprises five areas for consideration: study design, training of intervention providers, treatment delivery, treatment receipt and enactment.^24^ While this model was developed for psychological interventions, it has been adopted in rehabilitation-focused research.^25 26^

### Randomisation

Based on clinical data, we estimate to recruit 6–7 participants per site in an 18-month period. We aim to recruit a total of 32 participants, 16 per group (block randomisation), consistent with the number of participants suggested for an exploratory pilot RCT,^27^ increased to allow for participants’ withdrawal rate of up to 25%.

Randomisation into one of two groups will be completed through an independent automated process via a concealed allocation through the Research Electronic Data Capture (RedCap),^28^ a secure, web-based application designed to support research data capture. All sites have access to RedCap and will use the same randomisation module (central randomisation). Randomisation sequence was generated by an independent statistician not involved in the trial and without knowledge of the trial details. The sequence was imbedded into RedCap by a team member not involved in recruitment, screening or treatment of participants. Outcome assessors and data analysts will be masked to the group allocation of the individual participants.

### Blinding

Trained outcome assessors and data analysts will be masked to the group allocation of the individual participants. Due to the nature of the intervention, the parents and therapists providing intervention visits cannot be masked to group allocation. Baseline data will be collected prior to randomisation (T0).

#### Training of intervention providers

##### Therapists

The therapists involved in liaising with the families and providing the VISIBLE intervention visits during the study period are all registered occupational or physiotherapists (Australia), licensed physical or occupational therapists (USA, Georgia) or neurodevelopmental therapists (Italy), who specialise in development across multiple domains including vision. All VISIBLE therapists have taken part in VISIBLE intervention-specific training. Training consisted of a 2-day face-to-face theoretical and practical course. Intervention and vision assessment manuals were developed with video examples of vision assessments.

### Treatment delivery

#### Home visits minimum dosing

The visits are planned to be completed once every 2 weeks until 12 months CA, for a minimum of 6 months duration, if the infant is recruited at the end of the eligibility period at 6 months CA. Completion of 10 visits (80% of the intervention target dose) within 6 months is required for adherence to the VISIBLE trial. Should the infant/family be unavailable for completing at least 10 visits prior to 12 months 30 days, the visits will continue via the principle of intention to treat (ITT) until the minimum number (10) of home visit sessions is completed.

#### Supporting the parent–infant relationship and parent mental health

The intervention will be conducted in a manner that promotes sensitive and responsive parenting.^29^ If concerns regarding parental mental health or the parent-infant relationship are identified—either through therapist-parent interactions or based on parental scores on the Depression Anxiety Stress Scale (DASS)^30^ or the Emotional Availability Self-Report Scale (EA-SR),^31^ available referral and services options will be discussed with the parent.

### Treatment receipt and enactment

#### Delivery and fidelity

The fidelity of the VISIBLE approach was illustrated using case studies. Throughout the study duration, VISIBLE therapists will receive continued training and peer support for the provision of programme from investigators with a speciality in vision intervention to ensure the intervention is carried out with fidelity.

As previously described,^32^ study therapists will video-record all home visit sessions, with recordings uploaded to a central server at the Queensland site. For each therapist delivering each intervention, the first two home visits will be independently reviewed by two study investigators at the Queensland site using the Provider Delivery Observational Checklist. Thereafter, a random selection of recordings will be reviewed throughout the study, such that approximately 10% of each therapist’s intervention sessions are evaluated using the same checklist. An 80% compliance threshold for checklist items will be applied; deviations beyond this threshold will prompt feedback to the study therapist’s local site supervisor. The supervisor and study therapist will then jointly review the feedback and identify strategies to reduce further deviations from the study protocol.

To monitor and enhance treatment receipt, study therapists will employ a range of strategies. Worksheets designed to support parents’ delivery of the intervention will be provided, including guidance on preparing for play sessions, prompts regarding optimal timing and environmental setup for play, and tools to encourage parent reflection on their play sessions with their child. During structured home visits, therapists will ask parents to demonstrate the intervention activities they have been practising with their child, as well as the materials used for goal-directed activities. Therapists will provide feedback and highlight the child’s progress.

As the child advances toward established goals, therapists will demonstrate subsequent stages or goals of therapy and discuss features of toys, objects or environmental setups that may elicit the targeted behaviours. Coaching strategies will be used to support families in identifying toys and objects available within their own environment that could be incorporated into therapy activities. Collaborative discussions will help therapists determine whether parents understand the upcoming therapy goals. Families may also video-record their child during sessions as a prompt to support intervention delivery between therapist visits.

Parents will be interviewed independently when their infant is at baseline, midway through the intervention period and at 12 months corrected age. These interviews will explore parents’ responses to the intervention requirements, the feasibility of intervention delivery within their family context and their perceptions of changes observed in their child. Interviews will be conducted following completion of the Pediatric Rehabilitation Intervention Measure of Engagement, which was developed to capture parents’ perspectives on their own engagement in their child’s therapy.

The interview will follow parent’s completion of the Pediatric Rehabilitation Intervention Measure of Engagement, developed to gain parent’s perspective on their own engagement in their children’s therapy.^33^

### Statistical methods

Study assessments will be completed prior to randomisation (T0), prior to the initiation of the intervention (T1), and after completion of the intervention period at the 12-month follow-up (T2).

#### Feasibility and acceptability evaluation

Feasibility of recruitment process will include number of infants referred and eligible and proportion subsequently recruited. Parent engagement will be measured on the Pediatric Rehabilitation Measure of Engagement-General (PRIME-G).^33^

Adherence to the intervention will be assessed by documenting the number of withdrawals, retention rate and number completing the minimum number of intervention visits.

Acceptability will be measured by considering reasons for not consenting to participate in the RCT, intervention adherence through parent diaries and a post-intervention questionnaire to assess satisfaction and parent perceptions of the intervention using the Canadian Occupational Performance Measure (COPM). Parent enactment of delivery of the intervention with their child in the home will be assessed from parent-recorded videos and assessed using a fidelity checklist.^34^

#### Primary outcome measure

The vision ability of infants at baseline (T0) and 12-month follow-up (T2) (primary outcome measure) will be determined with vision function assessments.

Visual acuity assessment: Teller Acuity Cards (half set, consisting of 8 cards) will be used in modulated gratings (between 0.23 and 26 cycles/cm) to measure maximum acuity. Testing distance of 38 cm is used for infants 0–6 months allowing for spatial frequencies measurement. This wide range is appropriate for visual acuity measurement in infants with low vision.

Infant Battery for Vision will be used.^35^ It consists of a set of standard assessments, testing:

- Spontaneous visual behaviour.
- Fixation.
- Oculomotor movements (nystagmus, strabismus).
- Visual primary perception.
- Visual fields* using the confrontation method.
- Contrast sensitivity (Hiding Heidi cards).
- Visual adult-infant interaction (face recognition, response to facial expressions).

The detailed explanation regarding the performance of the visual function assessments is provided in the study-specific written manual and video recording examples formats.

Assessments of vision will also include:

- Ophthalmological assessment, including eye examination, assessment of refractive error, eye deviation and oculomotor behaviour.
- Near detection scale (Sonsken)^36^: a 10-point scale ranging from no light awareness (0) to 0.1 cm ‘lure’ (9) according to visual fixation on diminishing size lures at standard to near distance (30 cm).
- The Preverbal Visual Assessment (PreViAs), a simple 30-item parent questionnaire, will be used for screening visual abilities (visual attention, visual communication, visual-motor coordination and visual processing).^37^ The PreViAs will be used for screening visual abilities (visual attention, visual communication, visual-motor coordination and visual processing).

#### Secondary outcome measures

The Peabody Developmental Motor Scales, Second Edition (PDMS-2)^38^ at T1, T2 is a standardised and normed scale for children aged from birth to 6 years and has been validated for use as a discriminative measure. Infant primary motor outcomes at 12 months CA will be assessed using the PDMS-2, a standardised, norm-referenced measure used to evaluate the gross and fine motor development of children aged birth to 6 years.

The Bayley Scales of Infant and Toddler Development, Third Edition (BSID-III)^39^ at T2, a standardised and norm-referenced assessment of cognitive, motor, language and social-emotional development of infants and toddlers 0–3 will be assessed using the gold standard Bayley Scales of Infant and Toddler Development, Third Edition. These assessments will be supplemented by parent report on the norm-referenced forms.

The COPM^40^ at T1, T2 is used to assist parents in setting and prioritising goals and measuring parent-perceived change of their infant’s performance of the goal and own satisfaction with progress. The semi-structured initial interview to establish the goals will focus on what aspects of the infant’s vision behaviour the parent wants or needs to see improved.

Pediatric Evaluation of Disability Inventory-Computer Assisted Test (PEDI-CAT)^41^ at T1, T2 is a parent-reported measure of their child’s independence in self-care, mobility and social function that has been analysed in both children with disabilities and those with typical development. Raw scores will be converted to standardised scores using normative data (0–100) to measure change in function. Developmental outcomes in self-care, mobility and social function will be assessed at 12 months CA using the PEDI-CAT, a standardised, norm-referenced assessment of independence in self-care. The instrument measures functional outcomes across four domains: daily activities, the ability to perform living skills; mobility, the ability to move around the home and in the community; and social/cognitive, the ability to participate and effectively engage in social situations. Responsibility, the fourth domain, will not be assessed in this study.^42^ The PEDI-CAT has good discriminant validity in CP populations, between children with and without disability, and demonstrates concurrent validity with the Wee-FIM (Functional Independence Measure for Children) in children with brain injury and developmental disabilities.^43^ The test is valid, reliable and responsive in this population, takes 10–15 min to complete and requires no formal training.

DASS-21^30^ at T1, T2 is a 21-item questionnaire that assesses symptoms of depression, anxiety and stress in the parent/caregiver.

Emotional Availability Scales (EAS) and Self Report^31^ at T1, T2 is based on a naturalistic observation of a parent-infant interaction in the family’s home to measure parental sensitivity, parental structuring, parental non-intrusiveness, parental non-hostility, child responsiveness and child involvement, and a global relationship quality rating. The video will be 15 min long. Parents will be asked to video the interaction, or telemedicine video recording will be used. The EAS will be assessed by an independent rater trained in scoring the EAS. The EAS self-report is a 32-item parent-report measure of emotional availability within the parent-child relationship.

The Infant Toddler Quality of Life Questionnaire (ITQOL)^44^ at T1, T2: ITQOL is a 103-item parent-reported questionnaire of quality of life for infants and toddlers aged 2 months to 5 years, and measures quality of life across physical, mental and social well-being. For each of the 10 concepts (physical functioning, growth and development, bodily pain, temperament and moods, general behaviour, getting along, general health perceptions, change in health, and parental impact: emotional/time/family activities/family cohesion), item responses are scored, summed and transformed to a scale from 0 (worst health) to 100 (best health).

Clinical Brain Structure and Neuroplasticity measures. When possible, brain MRI will be acquired on 1.5T or 3T scanners in the neonatal period (as part of recruitment) (T0) and at 12-month follow-up (T2). All attempts will be made to complete a natural sleep MRI with either (i) feed and wrap methods in the neonatal period and/or (ii) at 12 months CA the implementation of a desensitisation programme to familiarise the child to the scanner noises so that sleeping scans can be performed. In addition to the recommended clinical MRI, we will acquire high-resolution structural images and multi-shell high angular resolution diffusion-weighted images (high angular resolution diffusion imaging (HARDI) and neurite orientation density and dispersion imaging (NODDI)).^45^ These additional research sequences will take approximately 15 min and will be acquired at the same time as the recommended clinical scan. Families who do not wish for their child to have an MRI around 12 months CA can participate in the study without performing the brain MRI scan.

All assessment tools and instruments intended for use by clinical researchers in this study are available in English. Additionally, tools requiring parental completion, including the DASS-21, ITQOL and EAS-R, are available in each local language (English, Georgian and Italian) to ensure accessibility and accurate data collection across study sites.

### Data analysis plan

A multisite RedCap programme database will be used for this study. Study personnel at each site will enter the data onto RedCap, a secure web platform for the construction and management of surveys and online databases. All the data of the same patient will be collected under an ID number. Videos of sessions will be taken to ensure fidelity of intervention visits provided by the study therapists.

All measures will be undertaken to avoid missing data for the primary outcome. Rescheduling of appointments for assessments will be done if the data collection cannot be completed in one appointment. Should vital data be missing, use of all available data will be used, as recommended through methods such as multiple imputation and likelihood-based analysis.

Statistical analysis will be conducted using SPSS following standard methods for RCTs. Intention-to-treat analysis will be applied to minimise inflation of treatment effects, consistent with Consolidated Standards of Reporting Trials (CONSORT) guidelines.^46^ Descriptive statistics will be used to summarise baseline characteristics and outcome measures for both treatment groups, with continuous variables reported as means and SDs (if normally distributed) or medians and IQRs (if skewed), and categorical variables as frequencies and percentages. Normality of continuous data will be assessed using the Shapiro-Wilk test and visual inspection of histograms and quantile-quantile (Q-Q) plots. If data are normally distributed, intervention efficacy will be tested using general linear models, specifically Analysis of Covariance (ANCOVA), adjusting for baseline values. If substantial skewness is observed and not resolved through transformation, non-parametric alternatives such as the Mann-Whitney U test for between-group comparisons will be used. Appropriate adjustments for multiple comparisons will be applied where necessary.

Brain MRI analysis will include classification and semi-quantitative assessment of brain structure by a paediatric neurologist masked to clinical history. In term-born infants, structural MIRs (sMRIs) will be classified according to the Hammersmith Term Hypoxic Ischaemic Encephalopathy (HIE) patterns.^47 48^ Basal ganglia and thalamic (BGT) lesions will be classified as: (i) mild, focal subtle abnormalities with normal appearance of the posterior limb of the internal capsule (PLIC); (ii) moderate, multifocal lesion with equivocal or abnormal signal intensity within the PLIC; (iii) severe, widespread abnormalities involving all BGT structures and PLIC.^49 50^ White matter (WM) changes are classified as: (1) mild, periventricular WM changes difficult to differentiate from normal appearances and therefore not classified as abnormal; (2) moderate, small focal lesions without loss of grey matter/WM differentiation; and (3) severe, larger areas of abnormality with loss of grey matter/WM differentiation, consistent with infarction.^51^

MRI pattern will be described as: (1) pattern 1: BGT lesions with severe WM abnormalities; (2) pattern 2: BGT lesions with mild or moderate WM abnormalities; (3) pattern 3: only thalamic lesions; (4) moderate WM lesions and (5) normal or mild WM changes in an infant aged 5 days.^52^ This semi-quantitative scoring system is used to assess the hemispheres, subcortical structures, corpus callosum and cerebellum with particular attention to the visual pathways. In children aged >12 months CA with early brain injury, the structural MRIs will be assessed using the qualitative measure of the extent of brain lesion based on the Fiori scale.^53^ The extended version of the deep grey matter area will be applied^54^ (the detailed involvement of the thalamic nuclei and globus pallidus and/or putamen will be assessed). For sMRI, the three orthogonal T2-weighted images (T2W) will be combined into a single high-resolution T2W MRI using image super-resolution.^55^ This allows the high-resolution T2W images to be segmented into tissue types and regions of interest using the developing Human Connectome Project (dHCP) structural pipeline, as^56^ well as generate measures of the cortical surface using a manually annotated neonatal atlas.^57 58^ Region-of-interest and voxel-based analysis will be calculated and their association with vision and motor skills will be investigated.

Where diffusion MRI (dMRI) is available, the analysis would provide quantitative information regarding the white matter microstructure using the fractional anisotropy (FA), fibre density and fibre orientation (tractography). Fractional anisotropy and mean diffusivity (MD) will be estimated using maps of diffusion tensor imaging (DTI) metrics and intracellular volume fraction, isotropic volume fraction and orientation dispersion would be calculated using NODDI metrics. Cortical and subcortical areas of interest will be demarcated using the age-appropriate atlases and the summary of DTI measures will be calculated. Estimation of fibre orientation distribution (FOD) and fibre densities of young-appearing and older-appearing white matter will be done using multi-shell multi-tissue constrained spherical deconvolution for neonates in MRtrix.^59 60^ Whole-brain voxel-based analysis of FA and MD would be performed using tract-based spatial statistics.^61^

### Ethics and monitoring

This study was approved by the Ethics Committee at the Children’s Health Queensland Human Research Ethics Committee, reference number HREC/18/QRCH/83. The research will be conducted in accordance with the Helsinki Guidelines. Institutional oversight ensures participant safety and data integrity, with adverse events managed locally and reviewed as needed. Standard oversight by institutional review boards and site investigators will be used to ensure participant safety and data integrity, with any solicited or spontaneously reported adverse events or unintended effects of trial interventions or conduct managed locally by these bodies and discussed during partner team meetings if necessary.

### Dissemination

Dissemination of findings will be through peer-reviewed publication of study results, plain language summaries, newsletter feedback and presentations at key national and international conferences. We will discuss findings with relevant consumer groups and seek their involvement with future trials.

### Patient and public involvement

It was not appropriate or possible to involve patients or the public in the design or conduct at this stage. Reporting or dissemination has not taken place yet, as this is a protocol.

## Discussion

This paper outlines the design and background for a pilot feasibility RCT comparing VISIBLE (Vision Intervention for Seeing Impaired Babies through Learning and Enrichment) to standard of care for infants with severe cerebral visual impairment and high risk of cerebral palsy. It addresses an urgent gap in rehabilitation interventions for infants with brain damage and severe vision impairments. Vision-aware early intervention is innovative in that the programme is applied within the infant’s natural environment and provided by the parents with support from the multidisciplinary team in the form of fortnightly home visits (or Zoom sessions for remote families). The potential significance of the project lies in a model of environmental adaptations to support the overall development of infants with severe CVI and motor difficulties through vision-focused modification.

## Supplementary material

10.1136/bmjopen-2025-114567 online supplemental file 1
