# Supplementary material for: Vision Intervention for Seeing Impaired Babies: Learning through Enrichment (VISIBLE) – protocol of a feasibility pilot randomised controlled trial
Source: BMJ Open. 2026 Apr 20;16(4):e114567. doi: 10.1136/bmjopen-2025-114567 (PMC13110617; doi:10.1136/bmjopen-2025-114567)
Supplement: online supplemental file 1 [file bmjopen-16-4-s001.pdf]

PARTICIPANT INFORMATION STATEMENT AND CONSENT FORM – PARENT

You do not have to agree to participate, it is ok to say no – this study is entirely voluntary.

Project Number: HREC/18/QRCH/83

Title of Project: VISIBLE: Vision Intervention for Seeing Impaired Babies through Learning and Enrichment.

Chief Investigators: Professor Roslyn Boyd, A/Professor Andrea Guzzetta, Professor Iona Novak, Dr Cathy Morgan, Dr Alison Salt, Dr Catherine Elliott, Professor Glen Gole, Dr Swetha Philip, Professor Nadia Badawi, Professor Stephen Rose, Dr Jurgen Fripp, Dr Kerstin Pannek, Dr Susan Greaves, Dr Roslyn Ward, Professor Rodney Hunt, Professor Russell Dale, A/Professor James Elder, Jessica Tibbs, Anya Gordon, Dr Lizelle Weber, Dr Margot Bosanquet, Dr Marnie Fraser, Dr Peter Schmidt, Dr James Smith, Ms Katie Geering.

Thank you for taking the time to read this Information Statement.
This information statement and consent is 8 pages long. Please make sure you have all the pages.

For people who speak languages other than English:
If you would also like information about the research and the Consent Form in your language, please ask the person explaining this project to you.

You and your child are invited to participate in a Research Project that is explained below.
What is an Information Statement?

These pages contain information about a research project we are inviting your child to take part in. The purpose of this information is to explain to you clearly and openly all the steps and procedures of this project. The information is to help you to decide whether or not you would like your child to take part in the research.

Please read this information carefully. You can ask us questions about anything in it. You may also wish to talk about the project with others e.g. friends or health care worker. Once you have understood what the project is about, if you would like your child to take part please sign the consent form at the end of this information statement. You will be given a copy of this information and consent form to keep.

What is the Research Project about?
This project includes families of infants with brain injury that occurred during pregnancy or around the time of birth which affected their motor and vision functions. In these situations, early therapeutic activities and treatments are recommended to optimize development.

The study has to do with rehabilitative intervention starting at 3-6 months + 29 days corrected age (C.A.) and lasting up to 12 months C.A. called VISIBLE. It includes a home-based therapeutic

program supported by therapists that actively engages parents in helping their babies develop. VISIBLE will include parent training and fortnightly home or telehealth visits.

This study is about testing whether VISIBLE makes a difference in the functional abilities of infants diagnosed as at risk of having cerebral palsy and vision impairment. It is also about finding out what you think of VISIBLE. Your active participation in refining VISIBLE is highly valued.

Can I participate?
You can participate in this study if you are the parent (including adoptive, step-parent or legal guardian) of an infant (currently under 6 months + 29 days C.A.) who has been diagnosed with, or is at risk of, cerebral palsy and has a vision impairment. This study is testing a parent supported home intervention, so you need to

- Allow 1 or 2 medical professionals (therapists) to come to your home fortnightly for a 90-minute visit (or have reliable internet connection and a computer device to complete these sessions over a video call).
- Be willing to discuss with the therapist/s ways that you (parents) can play and interact with your baby in a way that supports their development.
- Be willing to review study-specific information given to you in the form of a Parent Booklet.
- Be willing to apply the overall suggestions for play and interaction with your infant, and engage in specific play activities with your infant each day for about 30 minutes

What will participation involve?
If you choose to participate in this study you will be randomly (like tossing a coin) allocated to one of two groups: intervention or standard care. The standard care group will continue with the local therapy services and participate in the assessments for the study.

The intervention group will receive therapy services including the study specific activities at home that will be completed by parents/caregivers (taught to parents/caregivers by the research team therapists). This allows us to identify whether there are any benefits to the parent and infant participating in the parent-led home activities.

You will need to complete several assessments during the study period.

The assessments for this study include:

- **Neuroimaging.** We will also ask whether you wish to give consent for your child having Magnetic Resonance Imaging (MRI) of the brain when he/she is 12 months C.A. We would like to do the MRI to compare changes over time from an MRI your infant may have had earlier in life and we would also like to look at whether children's brains respond differently to the two different interventions approached. The MRI will be completed at the Neuroimaging department of the Queensland Children's Hospital.

If your child has had an MRI in the first 3 months of life we will ask your child's treating doctor to provide a copy of the MRI after your child is referred to us. If you give consent for your child to have an MRI at 12 months C.A. the scanner will take pictures of your child's brain using magnetic and radio waves. No X- rays are used. MRI brain scans are safe and routinely done for children who have a suspected brain injury to determine the nature of the brain injury.

Your child will be positioned on a comfortable pillow in the scanner and monitored over the scan time (approximately 30 minutes). Your child will find in difficult to stay still in the noisy environment of the MRI, so there are two options: (i) your child can be desensitized to the scanner sounds by learning to go to sleep at home with the scanner noise playing in the back ground, and then the MRI may be able to be completed while your child is asleep; (ii) the MRI can be performed under a General Anaesthetic.

The formal report of the scan by the Radiologist will be given to you and the results will be forwarded to your child's treating Paediatrician or Rehabilitation Doctor. Although the MRI is

offered and may provide helpful information, your child can participate in the study even if you choose not to have the MRI scan. Also, you can choose not to have the MRI scan at 12 months C.A., even if you give consent for the MRI now.

- **Ophthalmologic (eye) exam**: We will review your baby's eye exam results and complete additional vision abilities tests with your baby. These additional tests will be behavioural, this means we will place cards and objects in front of your baby's vision field to understand how much and how well she/he sees.

- **General Movements Assessment**: This assessment will be completed when your infant starts in the study between 3 and 6 months of age. We will choose a time when your infant is awake and is between feeds. Your infant will be placed on his/her back, in a nappy. We will then videotape your infant's natural spontaneous movements for about 5 minutes. It will last about 10 minutes altogether. A certified assessor will analyze the quality of your infant's movement from the videotape at a later time.

- **Parent questionnaires:** These questionnaires will ask you about your family, feelings and wellbeing, as well as your child's behavior and your own relationship to your child. These will not take more than 15 minutes to complete. Other questionnaires will ask you about your opinion of your infant's vision abilities, your baby's interaction and playing style and abilities, and your view of their quality of life. All of the assessments should not take more than 30 minutes to complete. Online and paper versions will be available.

- **A semi-structured parents' interview:** The Canadian Occupational Performance Measure helps the medical team and parents to establish goals which will focus on what aspects of the infant's vision behaviour that you (the parent) wants or needs to see improved.

- **Neurological Assessment:** The Hammersmith Neurological Examination is done by a therapist/doctor who is a Paediatrician, Rehabilitation Specialist or Neurologist. This test lasts between 10 and 20 minutes and assesses the neurological function of the infant. The doctor will interact with your baby, hold them, and check his/her reflexes.

- **Bayley III Developmental Assessment:** This assessment will be completed at 12months C.A.to assess cognitive development, language and motor abilities. The Bayley III consists of a series of play-based activities with infants and will take about 60 minutes to do.

- **Peabody Developmental Motor Scales - 2 edition (PDMS-2):** This assessment will be completed by a therapist and will assess your child's motor skills.

- **Video Recording:** A 20-minute unstructured interaction between you and your child to get a flavour of how the two of you interact together. We will assist you with this recording and it can be done via a video call (Zoom) or you can use your own recording device. We will help you record and transfer the interaction in an entirely secure way.
  If both parents are participating, then both will be asked to complete the interaction video assessments. We will also ask you to share your thoughts on VISIBLE.

How will participation benefit me?
It is recommended to start intervention early with babies with neurologic differences. Based on our previous work we believe there is a potential benefit to infants in both groups. However, there is no guarantee that you or your child will benefit from this study. You will play a valuable role in evaluating the usefulness of the intervention program.

Is there likely to be a benefit to other people in the future?
We hope that the results of our project will help infants with early brain abnormalities and their families in the future. With your help, we hope to understand the usefulness of the program and if proven to make a difference, to develop VISIBLE into an effective program accessible to parents of children with a diagnosis, or at risk, of cerebral palsy together with vision impairments across the world.

Assessments schedule for the study, for both groups				
	Eligibility	3-6 mths + 29 days at enrolment	12mths end of study	Approximate time (min)
Visual acuity assessment, including Ophthalmologic exam (clinical)	•	•	•	30
Neuroimaging (MRI)	• (clinical review in the neonatal period)		• at 3T under GA (optional)	45
General Movements	•			10
Neurologic Assessment (HINE)		•	•	15
Parent questionnaires		•	•	30
Video of parent-child interaction EAS		•	•	20
Semi-structured parent interview COPM		• (Intervention group only)	• (Intervention group only)	20
Peabody Developmental Motor Scales-2		•	•	30
Bayley Scales of Infant and Toddler Development III			•	60

Are there any risks to participation?
We do not expect that there will be any risks or side effects from participating in this study, as it is totally non-invasive and based on therapy approaches that are used in clinical practice.

There are no additional risks for your child with these measurements (including Magnetic Resonance Imaging, MRI) over and above that experienced during routine clinical examinations with their doctor. All procedures are frequently used for both clinical and research purposes.

You are under no obligation to consent to your child having a brain MRI scan. There are some risks associated with your child having a general anaesthetic to undertake the MRI. In ambulant children with Cerebral Palsy with no other major medical conditions (no uncontrolled seizures) then the risk associated with General Anaesthesia is a 1% chance of some complication. The referring doctor will discuss this with you in relation to your infant.

What are the possible discomforts and/or inconveniences?
The therapy program will be performed at home by you or one of your infant's other caregivers. The amount of therapy we ask you to complete each day is tailored to take into account your infant's development. Therapists will help you work out ways of incorporating the therapy into your daily routine, making it part of your play time with your child.

The MRI scanner is noisy, so protective earmuffs will be placed over your child's ears during the scan. The assessment appointments will be planned to minimize any inconvenience to you by predominantly occurring at home. The appointment at 12months C.A. at the hospital will be organized to coincide with any other appointment that you may have at the hospital. We will pay for the cost of parking your car at the hospital during these visits.

What happens if something abnormal or unexpected is found in my child's MRI scan?
In this study, we will take a number of pictures of your child's brain (using MRI), or will review MRI pictures that have already been taken. After your child's scan, a specialist will review these

pictures. This will not be done on the day of the scan. In children with cerebral palsy there is a high chance of finding an abnormality on the brain scan. Often this finding will help to explain the cerebral palsy. There is also the possibility that the scan will show up something in your child's brain that we had not expected. If this happens, we will arrange for you to meet with a medical professional who can explain the findings to you. If any of the results of the scan are distressing for you, we will offer you counseling with specially trained staff.

Will my information be confidential?

Yes! All information that we collect from you, including your questionnaires, videoed parent-child interaction and your feedback will be stored in a confidential manner on a password-protected University of Queensland secure server. Records including recordings will be retained in accordance with the obligations under the *Public Records Act 2002* and associated State policies, retention and disposal schedules and other official advice issued by the Queensland State Archives' State Archivist https://www.qld.gov.au/dsiti/qsa. De-identified, collated data will be shared with other researchers and clinicians at conferences and through publications.

What if I change my mind?

You do not have to take part in this research project. Your participation will not affect any treatment that your child receives. If you do agree to participate, and change your mind at a later date, you are free to withdraw from the study at any time without any negative consequence.

Research Contacts:

Chief Investigators

1. Professor Roslyn Boyd is the Scientific Director of Queensland Cerebral Palsy and Rehabilitation Research Centre. She has clinical and scientific expertise in working with infants and children with cerebral palsy and their families.
2. A/Professor Andrea Guzzetta is the Head of Infant Neurology and Stella Maris Infant Lab for Early intervention. His research focuses on the development of different functions (motor, visual) with an overall aim of improving early intervention.
3. Professor Iona Novak is a Paediatric Occupational Therapist and Head of Research at the Cerebral Palsy Alliance. She will oversee the conduct of the study in New South Wales.
4. Dr Cathy Morgan is a Paediatric Physiotherapist. She will assist with overseeing the conduct of the study in New South Wales.
5. Dr Alison Salt is a Consultant Paediatrician at Perth Children's Hospital and will be responsible for referral of infants to the study and conducting the eye assessments in Western Australia.
6. Professor Catherine Elliott is a Paediatric Occupational Therapist and Professor of Allied Health Research, at the Perth Children's Hospital. She will oversee and manage the study in Western Australia.
7. Professor Glen Gole is a Consultant Paediatric Ophthalmologist, Department of Ophthalmology at The Queensland Children's Hospital. He will be responsible for the interpretation and analysis of the vision assessments.
8. Dr Swetha Philip is a Paediatric Ophthalmologist and will be responsible for recruitment, data collection and analysis.
9. Professor Nadia Badawi is the Medical Director of the Grace Centre for Newborn Care, Children's Hospital at Westmead and will coordinate the study at this site.
10. Professor Stephen Rose is a Scientific Leader at CSIRO and oversee the MRI data collection.
11. Dr Jurgen Fripp is a Senior Research Scientist at CSIRO and will assist with the MRI data analysis.
12. Dr Kerstin Pannek is a Research Scientist at CSIRO and will assist with the MRI data analysis.
13. Dr Susan Greaves is a Senior Occupational Therapist at the Royal Children's Hospital in Melbourne who will oversee the conduct of the study in Victoria.
14. Dr Roslyn Ward is a Senior Research Fellow at Perth Children's Hospital who will assist with overseeing the conduct of the study in Western Australia.
15. Prof Rodney Hunt is a Neonatal neurologist at the Royal Children's Hospital in Melbourne who will be responsible for referring children for the MRI scans.
16. Professor Russell Dale is a paediatric neurologist at the Children's Hospital at Westmead who will be responsible for referring children for the MRI scans.

17. A/Professor James Elder is a paediatric ophthalmologist at Royal Children's Hospital in Melbourne who will be responsible for referral of infants to the study and conducting the eye assessments in Melbourne.
18. Jessica Tibbs is an Occupational Therapist at Cairns Hospital who will oversee the conduct of the study in Cairns, Anya Gordon is a Physiotherapist Therapist at Cairns Hospital who will oversee the conduct of the study in Townsville,
19. Anya Gordon is a Physiotherapist Therapist at Cairns Hospital who will oversee the conduct of the study in Townsville.
20. Dr Margot Bosanquet is a Paediatrician in Townsville who will assist with referral and assessments.
21. Dr Lizelle Weber is the Director of the Special Care Nursery at Sunshine Coast University Hospital who will assist with referral and medical advice for the early care and diagnosis of infants.
22. Dr Marnie Fraser is a Paediatrician in Cairns who will assist with referral and assessments.
23. Dr Peter Schmidt is the Director of Neonatology at Gold Coast University Hospital who will assist with referral and medical advice for the early care and diagnosis of infants.
24. Dr James Smith is a neonatal ophthalmologist at the Children's Hospital at Westmead who will assist with referrals and assessments.
25. Ms Katie Geering is an orthoptist at the Children's Hospital at Westmead who will assist with referrals and assessments.

Do you have any questions?
Please take the time to ask us any questions that you may have.
Study email: visible@uq.edu.au
Name: Prof Roslyn Boyd, Professor Cerebral Palsy and Rehabilitation Research,
The University of Queensland
Contact telephone: (07) 3069 7370 **mobile:** 0434 608 443

Ethics Contact:

Children's Health Queensland Hospital and Health Service Human Research Ethics Committee has approved this study (HREC/18/QRCH/83) Should you wish to discuss the study with someone not directly involved, in relation to matters concerning policies, information about the conduct of the study or your rights as a participant, or if you wish to make a confidential complaint, please contact:

CHQHHS Ethics Committee Coordinator:
Children's Health Queensland Hospital and Health Service
Human Research Ethics Committee
Level 7, Centre for Children's Health Research
62 Graham Street
South Brisbane QLD 4101; T: (07) 3069 7002

PARTICIPANT CONSENT FORM

Project Number: HREC/18/QRCH/83

Title of Project: VISIBLE: Vision Intervention for Seeing Impaired Babies through Learning and Enrichment.

Chief Investigators: Professor Roslyn Boyd, A/Professor Andrea Guzzetta, Professor Iona Novak, Dr Cathy Morgan, Dr Alison Salt, Professor Catherine Elliott, Professor Glen Gole, Dr Swetha Philip, Professor Nadia Badawi, Professor Stephen Rose, Dr Jurgen Fripp, Dr Kerstin Pannek, Dr Susan Greaves, Dr Roslyn Ward, Professor Rodney Hunt, Professor Russell Dale, A/Professor James Elder, Jessica Tibbs, Anya Gordon, Dr Lizelle Weber, Dr Margot Bosanquet, Dr Marnie Fraser, Dr Peter Schmidt, Dr James Smith, Ms Katie Geering.

I/We, ____

voluntarily consent to participate in the above titled Research Project explained to me by:

Mr/Ms/Dr/Professor ____

By signing this form, I state that:

- I/We have read the information statement for this study and I/we believe I/we understand the purpose, extent and possible effects of my/our involvement.
- I/We have had an opportunity to ask questions and I/we am satisfied with the answers I/we have received.
- I/We understand information collected will be stored confidentially and my/our identity will not be revealed.
- I/We understand that I/we can refuse to participate and can withdraw from this study at any time without any negative consequence. In particular, I/we understand that my/our participation will not affect my child's access to treatment.
- I/We understand that the purpose of this study is to pilot an innovative, in-home approach of parent delivered intervention activities with support from the research team therapist/s and that my/our active involvement, including critical feedback, is valued.
- I/We understand that in order to evaluate the new online parenting support package I/we will be asked to complete online questionnaires as well as record a parent-child interaction with my/our child three or four times during the study.
- **I/We consent for the study to access relevant data from my child's medical records and/or treating clinician ❑yes ❑ no**
- **I/We consent for the study to review my child's neonatal Brain MRI scan or Cranial Ultrasound that was performed in the first few months ❑ yes ❑ no**
- **I/We consent for my child to receive the Brain MRI scan ❑ yes ❑ no**
- **I/we consent for my child to participate in this research project ❑ yes ❑ no**
- **Have you and/or your child been involved in any other research studies? If yes, name of the study/studies: ❑ yes ❑ no**

  ____

OPTIONAL – Further Research

- I would like to be contacted about any future research trials that I may be eligible to participate in. (NB. full ethical approval would be sought by the research team and a new consent process undertaken) **❑ yes ❑ no**

Signature ____ **Date** ___________

Signature ____ **Date** ___________

(Signatures of two parents are required ONLY if two parents intend to participate in the course. All parties must date their own signature.)

I have explained this study and I believe that the participant/s understands the purpose, extent and possible effects of involvement.

Researcher's Signature ____ **Date** ___________
