# Supplementary material for: Vision Intervention for Seeing Impaired Babies: Learning through Enrichment (VISIBLE) – protocol of a feasibility pilot randomised controlled trial
Source: BMJ Open. 2026 Apr 20;16(4):e114567. doi: 10.1136/bmjopen-2025-114567 (PMC13110617; doi:10.1136/bmjopen-2025-114567)
Supplement: Supplementary file 2 [file bmjopen-2025-114567.reviewer_comments.pdf]

PEER REVIEW HISTORY

BMJ Open publishes all reviews undertaken for accepted manuscripts. Reviewers are asked to complete a checklist review form and are provided with free text boxes to elaborate on their assessment. These free text comments are reproduced below.

ARTICLE DETAILS

Title (Provisional)

Vision Intervention for Seeing Impaired Babies: Learning through Enrichment (VISIBLE): Protocol of a Feasibility Pilot Randomized Controlled Trial

Authors

Guzzetta, Andrea; Bancale, Ada; bedoshvili, anna; Bosanquet, Margot; Chorna, Olena; Corsi, Giulia; Del Secco, Sabrina; Elliott, Catherine; Fiori, Simona; Fripp, Jurgen; Gole, Glen A; Gordon, Anya; Harpster, Karen; Hunt, Rod W; Leishman, Shaneen; Mori, Rosalie; Morgan, Catherine; Novak, Iona; Pagnozzi, Alex Michael; Pannek, Kerstin; Philip, Swetha Sara; Rice, Melissa; Salt, Alison; Shannon, Bernadette; Schwartz, Terry; Tatishvili, Nana Nino; Tatishvili, Sofia; Boyd, Roslyn N

VERSION 1 - REVIEW

**Reviewer**	**1**
**Name**	**Pekçetin, Serkan**
**Affiliation**	**University of Health Sciences Turkey**
**Date**	**04-Feb-2025**
**COI**	**None**

Reviewer Comments on the Study:

The study analyzes the effectiveness of the VISIBLE intervention protocol on infants with cerebral palsy (CP) who are at risk of visual impairment. Below are my comments.

Abstract:
The expanded version of the VISIBLE intervention protocol should be mentioned first, followed by the abbreviation.

In the "Methods and Analysis" section, please include the specific instruments that will be used to evaluate the infants.

Background:
Page 6, Line 23: A more thorough literature review is required. To my knowledge, there

are additional studies that focus on CVI rehabilitation. Below are some relevant studies that should be included in your background:

Cemali, M., Pekçetin, S., & Akı, E. (2022). The effectiveness of sensory integration interventions on motor and sensory functions in infants with cortical vision impairment and cerebral palsy: a single-blind randomized controlled trial. Children, 9(8), 1123.
Fazzi, E., Micheletti, S., Calza, S., Merabet, L., Rossi, A., & Galli, J., Early Visual Intervention Study Group. (2021). Early visual training and environmental adaptation for infants with visual impairment. Developmental Medicine and Child Neurology, 63(10), 1180–1193.

Please revise the hypothesis without referring to the instruments used.

Methods:
Describe the intervention protocol in accordance with the CONSORT Checklist. Provide information about the language availability of the instruments, considering that this study is multicenter in nature.

**Reviewer**	**2**
**Name**	**Cemali, Mustafa**
**Affiliation**	**Trakya Universitesi**
**Date**	**09-Feb-2025**
**COI**	**None**

I think that the research is original and important in terms of its subject. However, it is seen that there are sections of the manuscript that need to be corrected and improved. The evaluation scales used in the summary method section are not specified, they should be added. No mention is made of intervention studies in the literature in the introduction section, the original value of this study cannot be understood without literature studies, therefore the research should be justified. It is unclear how the sample size was determined in the method section, a basis should be established so that the sample size is not insufficient, otherwise the reliability of the results may be questioned. Very brief information is given about the evaluation scales used, these scales should definitely be detailed. The interventions must definitely have studies on which they are based, these should be specified, it is not appropriate to create them according to the researchers' initiative. The original flow diagram of CONSORT, which is in the CONSORT guidelines but not in the manuscript, should be added. The Declaration of Helsinki should be made. Very superficial information is given in the data analysis section, and it is stated that ANCOVA will be used without checking whether normality is achieved or not. It should be stated how the normality test of the data will be performed and, accordingly, which descriptive categorical and numerical variables (such as mean, standard deviation or median) and tests for comparisons between groups will be used in cases where the data is normally distributed or not. In line with these suggestions, all areas of the manuscript should be reviewed and edited from beginning

to end. In particular, the level of evidence of the method section should be seriously increased.

I wish you good luck

Revision

1. The method section of the abstract does not explain which assessment scales will be used. This information should be added. The duration of the sessions should also be added. It should be stated how randomization was done and how many people will be in the groups. The method section of the abstract should be detailed.
2. In the introduction section, more general information is given. Studies in the literature are not mentioned. Studies in the literature should be mentioned, the difference between the current study and these studies should be revealed and the study should be justified.
3. In the introduction section, the specific effects of visual impairment on motor, cognitive and social development can be further detailed with studies in the literature.
4. In determining the sample size for the study, a power analysis should be performed using the G Power program or a similar study in the literature should be cited. When doing this, it is essential to specify what is being referenced. Otherwise, this may lead to limitations in interpreting the results of the study. Perhaps this sample size should only be considered within the scope of a pilot study.

5. Mettod; "İnclusion criteria i) Visual impairment (visual acuity values below 2 Standard Deviations (SD) for their corrected age), based on visual acuity assessment using Teller Acuity

Cards half set, consisting of 8 cards. They" Dahil etme kriterleri bölümündeki teller kartlarına ait bu bilgiye kaynak ekleyiniz.

6. After the statement that consent will be obtained for participants, add the statement "The research will be conducted in accordance with the Helsinki Guidelines."
7. A CONSORT flow diagram regarding the participants should be added. It should be stated what will be done at which stage of the study.

8. It is not clear which intervention the intervention plan was prepared based on. The source should be cited as a basis for the content of the intervention.
9. Very limited information has been provided about the evaluation scales to be used. The explanation of these scales should be clear and detailed so that researchers who read them can easily do it. Therefore, very short and superficially written scale explanations should be detailed. In addition, what the increase and decrease in scores mean and whether the validity and reliability tests have been done or not, and if so, information about the validity and reliability shift should be given.
10. The statistical analysis section should be detailed. It is not appropriate to declare that parametric analysis methods will be used without performing a normality test on the data. Priorities should be specified as to how the normality test of the data will be performed and which analysis methods will be used when the data is normally distributed or not. It should also be explained how descriptive data will be presented

between groups. It should also be detailed how randomization will be performed. 11. Data analysis; "Intention-to-treat analysis will be applied to minimize inflation of treatment effects consistent with CONSORT guidelines" Add source.

VERSION 1 - AUTHOR RESPONSE

Reviewer: 1
Dr. Serkan Pekçetin, University of Health Sciences Turkey
Comments to the Author:
Reviewer Comments on the Study:

The study analyzes the effectiveness of the VISIBLE intervention protocol on infants with cerebral palsy (CP) who are at risk of visual impairment. Below are my comments.

Abstract:
C: The expanded version of the VISIBLE intervention protocol should be mentioned first, followed by the abbreviation.

R: *This was included*

C: In the "Methods and Analysis" section, please include the specific instruments that will be used to evaluate the infants.

R: *We included the titles of the* ***Infant Battery for Vision****, and the Peabody Developmental Motor Scales 2 edition in the text of the abstract. Unfortunately listing each tool is not possible due to the character limit.*

Background:
C: Page 6, Line 23: A more thorough literature review is required. To my knowledge, there are additional studies that focus on CVI rehabilitation. Below are some relevant studies that should be included in your background: Cemali, M., Pekçetin, S., & Akı, E. (2022). The effectiveness of sensory integration interventions on motor and sensory functions in infants with cortical vision impairment and cerebral palsy: a single-blind randomized controlled trial. Children, 9(8), 1123. Fazzi, E., Micheletti, S., Calza, S., Merabet, L., Rossi, A., & Galli, J., Early Visual Intervention Study Group. (2021). Early visual training and environmental adaptation for infants with visual impairment. Developmental Medicine and Child Neurology, 63(10), 1180–1193.

R: *Thank you. We updated and expanded the background section accodingly.*

C: Please revise the hypothesis without referring to the instruments used.

***R:** We have revised the hypothesis section and removed each instrument named for the respective outcome measure.*

Methods:

C: Describe the intervention protocol in accordance with the CONSORT Checklist.

***R:** Thank you, we have restructured the Methods section.*

C: Provide information about the language availability of the instruments, considering that this study is multicenter in nature.

***R:** This statement confirmation has been included into the methods section. Thank you*

Reviewer: 2
Dr. Mustafa Cemali, Trakya Universitesi
Comments to the Author:

Reviewer Comment

I think that the research is original and important in terms of its subject. However, it is seen that there are sections of the manuscript that need to be corrected and improved. The evaluation scales used in the summary method section are not specified, they should be added. No mention is made of intervention studies in the literature in the introduction section, the original value of this study cannot be understood without literature studies, therefore the research should be justified. It is unclear how the sample size was determined in the method section, a basis should be established so that the sample size is not insufficient, otherwise the reliability of the results may be questioned. Very brief information is given about the evaluation scales used, these scales should definitely be detailed. The interventions must definitely have studies on which they are based, these should be specified, it is not appropriate to create them according to the researchers' initiative. The original flow diagram of CONSORT, which is in the CONSORT guidelines but not in the manuscript, should be added. The Declaration of Helsinki should be made. Very superficial information is given in the data analysis section, and it is stated that ANCOVA will be used without checking whether normality is achieved or not. It should be stated how the normality test of the data will be performed and, accordingly, which descriptive categorical and numerical variables (such as mean, standard deviation or median) and tests for comparisons between groups will be used in cases where the data is normally distributed or not. In line with these suggestions, all areas of the manuscript should be reviewed and edited from beginning to end. In particular, the level of evidence of the method section should be seriously increased.

I wish you good luck

Revision

C1: The method section of the abstract does not explain which assessment scales will be used. This information should be added. The duration of the sessions should also be added. It should be stated how randomization was done and how many people will be in the groups. The method section of the abstract should be detailed.

R: *The abstract has been changed. We now have the titles of the Infant Battery for Vision, and the Peabody Developmental Motor Scales 2 edition in the text of the abstract. The information abouit duration of sessions, randomization and group size have been included in the text.*

C2: In the introduction section, more general information is given. Studies in the literature are not mentioned. Studies in the literature should be mentioned, the difference between the current study and these studies should be revealed and the study should be justified.

R: A more thorough account of the literature has been added, underscoring the need for further studies that address the very early stages of development and that use gold standard designs (i.e. RCTs).

C3: In the introduction section, the specific effects of visual impairment on motor, cognitive and social development can be further detailed with studies in the literature.

R3: *We have expanded the paragraph, including a more detailed description of the effect of visual impairment on different aspects of infant development.*

C4: In determining the sample size for the study, a power analysis should be performed using the G Power program or a similar study in the literature should be cited. When doing this, it is essential to specify what is being referenced. Otherwise, this may lead to limitations in interpreting the results of the study. Perhaps this sample size should only be considered within the scope of a pilot study.

R4: *Indeed this is a pilot study as stated now also in the title. The sample size was consistent with this design and this was better clarified in the Randomization paragraph.*

C5: Methods; "İnclusion criteria i) Visual impairment (visual acuity values below 2 Standard Deviations (SD) for their corrected age), based on visual acuity assessment using Teller Acuity Cards half set, consisting of 8 cards. Include a source for this information in the inclusion criteria section.

R5: *The source has been added.*

C6: After the statement that consent will be obtained for participants, add the statement "The research will be conducted in accordance with the Helsinki Guidelines."

R6: *The sentence has been added.*

C7: A CONSORT flow diagram regarding the participants should be added. It should be stated what will be done at which stage of the study.
R7: *The diagram has been added.*

C8: It is not clear which intervention the intervention plan was prepared based on. The source should be cited as a basis for the content of the intervention.

R8: *The intervention was specifically designed for this project. The description has been changed by adding references to the principles and knowledge it was based on.*

C9: Very limited information has been provided about the evaluation scales to be used. The explanation of these scales should be clear and detailed so that researchers who read them can easily do it. Therefore, very short and superficially written scale explanations should be detailed. In addition, what the increase and decrease in scores mean and whether the validity and reliability tests have been done or not, and if so, information about the validity and reliability shift should be given.

R9: *We have included a description of all scales used, with specific references, with a level of detail consistent with space constraints.*

C10: The statistical analysis section should be detailed. It is not appropriate to declare that parametric analysis methods will be used without performing a normality test on the data. Priorities should be specified as to how the normality test of the data will be performed and which analysis methods will be used when the data is normally distributed or not. It should also be explained how descriptive data will be presented between groups. It should also be detailed how randomization will be performed.

R10: *The statistical paragraph has been changed to account for the reviewer's comments. Randomization details are reported in the "Randomization" section.*

C11. Data analysis; "Intention-to-treat analysis will be applied to minimize inflation of treatment effects consistent with CONSORT guidelines" Add source.

R11: *Reference has been added.*
